# Combination of apolipoprotein-A-I/apolipoprotein-A-I binding protein and anti-VEGF treatment overcomes anti-VEGF resistance in choroidal neovascularization in mice

**DOI:** 10.1038/s42003-020-1113-z

**Published:** 2020-07-16

**Authors:** Lingping Zhu, Mackenzie Parker, Nduka Enemchukwu, Megan Shen, Guogang Zhang, Qing Yan, James T. Handa, Longhou Fang, Yingbin Fu

**Affiliations:** 1 The Xiangya Hospital of Central South University, 87 Xiangya Road, Changsha Kaifu District, Changsha, Hunan China; 2 Department of Cardiovascular Sciences, Center for Cardiovascular Regeneration, Houston Methodist DeBakey Heart and Vascular Center, Houston Methodist, Houston, TX 77030 USA; 3 Cullen Eye Institute, Baylor College of Medicine, Houston, TX 77030 USA; 4 Rice University, Houston, TX 77005 USA; 5 The Third Xiangya Hospital of Central South University, 138 Tongzipo Road, Changsha Yuelu District, Changsha, Hunan China; 6 Wilmer Eye Institute, Johns Hopkins School of Medicine, Baltimore, MD 21205 USA; 7 Department of Cell and Developmental Biology, Weill Cornell Medical College, New York, NY 10065 USA

**Keywords:** Visual system, Macular degeneration

## Abstract

Many patients of choroidal neovascularization (CNV) are unresponsive to the current anti-VEGF treatment. The mechanisms for anti-VEGF resistance are poorly understood. We explore the unique property of the apolipoprotein A-I (apoA-I) binding protein (AIBP) that enhances cholesterol efflux from endothelial cells and macrophages to thereby limit angiogenesis and inflammation to tackle anti-VEGF resistance in CNV. We show that laser-induced CNV in mice with increased age showed increased resistance to anti-VEGF treatment, which correlates with increased lipid accumulation in macrophages. The combination of AIBP/apoA-I and anti-VEGF treatment overcomes anti-VEGF resistance and effectively suppresses CNV. Furthermore, macrophage depletion in old mice restores CNV sensitivity to anti-VEGF treatment and blunts the synergistic effect of combination therapy. These results suggest that cholesterol-laden macrophages play a critical role in inducing anti-VEGF resistance in CNV. Combination therapy by neutralizing VEGF and enhancing cholesterol removal from macrophages is a promising strategy to combat anti-VEGF resistance in CNV.

## Abstract (web summary)

By combining advanced age and laser photocoagulation, Zhu et al. establish a novel CNV model of anti-VEGF resistance. They show that lipid accumulation in old macrophages promote angiogenesis, and that apoA-I binding protein (AIBP) inhibits angiogenesis by reducing this lipid accumulation. They further show that the combination of AIBP/apoA-I and anti-VEGF treatment overcomes anti-VEGF resistance and effectively suppresses CNV.

## Introduction

Age-related macular degeneration (AMD) is the leading cause of irreversible blindness in the elderly. The projected number of people with AMD is 196 million in 2020 and 288 million in 2040, representing a substantial global burden on the healthcare system^1^. Choroidal neovascularization (CNV or wet AMD), which accounts for 10–20% of AMD, is responsible for 80–90% of blindness in AMD^2^. Although anti-VEGF therapies have revolutionized the treatment for CNV, many patients are unresponsive to anti-VEGF agents or experience a gradual loss of efficacy after repeated administrations over time^3^. For example, up to one-fourth of all treated patients, defined as non-responders, do not benefit from anti-VEGF therapy, with visual acuity deteriorating over time despite treatment^4^. 19.7%–36.6% of patients had active exudation after one year of regular 2.0 mg aflibercept treatments (VIEW 1 and VIEW 2 trials)^5^. The mean visual acuity gradually decreased during long-term follow-up with a pro re nata retreatment when patients exited from the MARINA or ANCHOR trial (SEVEN-UP Study)^6^. Thus, development of an effective therapy for anti-VEGF non-responders in AMD represents an unmet clinical need. The efforts to develop new treatments are hampered by poor understanding of the mechanisms underlying anti-VEGF resistance and the lack of suitable AMD animal models that exhibit anti-VEGF resistance. Previous studies have shown that macrophages have increased density and proliferative activity in response to bevacizumab treatment in surgically excised human CNV membranes^7^, suggesting that macrophages play a role in anti-VEGF resistance. Reduced cholesterol efflux in old macrophages promotes CNV formation^8^ and macrophage depletion inhibits experimental CNV^9,10^, further implicating macrophages as a key player in CNV pathogenesis. Finally, VEGF165 acts as a proinflammatory cytokine targeting monocytes, macrophages and leukocytes, in a positive feedback loop that involves endothelial cells to sustain pathological neovascularization process^11,12^. These studies prompted us to explore a new treatment strategy for CNV by targeting both VEGFR2 signaling and macrophages. We and others reported that the apolipoprotein A-I (apoA-I) binding protein (AIBP) enhances cholesterol efflux in endothelial cells and macrophages^13–15^, two cell types implicated in CNV pathogenesis. In endothelial cells, AIBP binds apoA-I containing high-density lipoprotein (HDL), and accelerates cholesterol efflux, which reduces lipid raft content and inhibits lipid raft-anchored VEGFR2 signaling, to thereby limit angiogenesis^13^. In macrophages, AIBP binds to toll-like receptor 4 (TLR4) in cholesterol-laden or inflamed macrophages/microglia to augment cholesterol efflux, normalize plasma lipid rafts, and decrease inflammation^14–16^. The ability of AIBP to target both hyperactive endothelial cells and cholesterol-laden macrophages makes it an ideal candidate to address the challenge of anti-VEGF resistance in CNV treatment.

In this study, we found that laser-induced CNV in mice with increased age showed increased resistance to anti-VEGF treatment, which correlates with the increased intracellular lipid accumulation in macrophages. The combination of AIBP/apoA-I and anti-VEGF treatment overcomes anti-VEGF resistance and effectively suppresses CNV.

## Results

### AIBP inhibits angiogenesis on retinal and choroidal endothelial cells

**Fig. 1:**
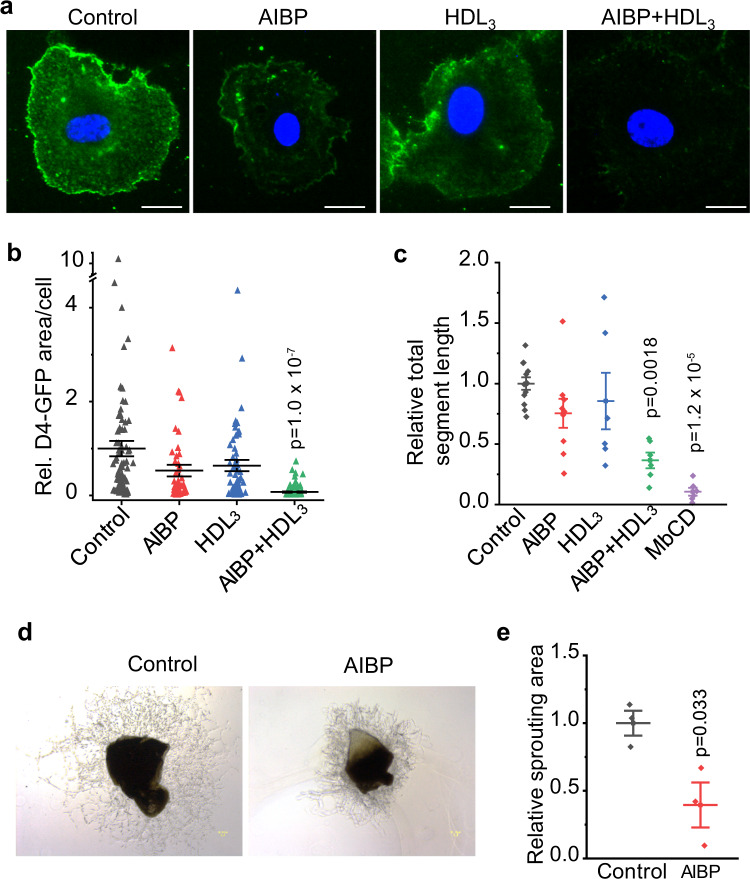
AIBP enhances cholesterol efflux and inhibits angiogenesis on retinal and choroidal endothelial cells. **a** Effect of AIBP and HDL_3_ on lipid rafts of HRMECs. Cells were stained for lipid rafts (green, D4-EGFP) and nuclei (blue, DAPI) after preincubation with control media, AIBP, HDL_3_, or AIBP plus HDL_3_. **b** Quantification of the area of lipid rafts per cell. *N* = 92, 54, 70, and 86 cells for control, AIBP, HDL_3_, and AIBP + HDL_3_, respectively. **c** Effect of AIBP and HDL_3_ on endothelial cell tube formation using the Matrigel-based assay. The total segment length of HRMEC tubes was quantified after various treatments. *N* = 12, 9, 6, 6, and 6 for control, AIBP, HDL_3_, AIBP + HDL_3_, and MβCD, respectively. Representative images (**d**) and quantification of microvascular sprouting area (**e**) after AIBP treatment. *N* = 3 explants per group. Data represent mean ± SEM in **b**, **c**, **e**. Statistical analysis was performed by one-way ANOVA with Tukey post hoc analysis (**b**, **c**) or two-tailed Student’s *t*-test (**e**).

AIBP and HDL together were shown to limit angiogenesis in human umbilical vein endothelial cells (HUVECs) by enhancing cholesterol removal^13^. The monkey choroidal EC line RF6/A and human retinal microvascular endothelial cells (HRMECs) are widely used as choroidal and retinal EC models, respectively. Since a recent study showed that the RF6/A cells do not exhibit key EC features^17^, we used HRMECs to investigate the role of AIBP in cholesterol efflux in retinal endothelial cells. HRMECs were incubated with control media, AIBP, HDL_3_ (a subfraction of HDL, which is an efficient cholesterol acceptor)^13^, or AIBP in combination with HDL_3_. The resulting cells were stained with recombinant D4-EGFP, which specifically binds cholesterol and has been used to monitor cellular lipid raft content^18^. The incubation with AIBP and HDL_3_ combination, but not either alone, markedly reduced lipid raft content on the plasma membrane (Fig. 1a, b). The data suggest that AIBP regulates cholesterol metabolism in HRMECs. We further examined the role of AIBP-mediated cholesterol efflux in HRMEC angiogenesis using the Matrigel-based in vitro tube formation model. As illustrated in Fig. 1c, AIBP and HDL_3_ co-treatment disrupted in vitro vascular tube formation by HRMECs. As a positive control, cholesterol depletion by methyl-β-cyclodextrin(MβCD)^19,20^, a detergent that sequesters free cholesterol, markedly inhibited angiogenesis. To explore the role of AIBP in choroidal angiogenesis, we used the ex vivo model of choroid sprouting, a reproducible model of choroidal angiogenesis^21^. Recombinant AIBP potently inhibited choroid sprouting (61% reduction, *p* = 0.035, Fig. 1d,e), consistent with the inhibitory role of AIBP in angiogenesis (Note, choroid explants were cultured 4 days in complete EGM-2 media, which contains HDL from bovine serum^22^).

### AIBP inhibits aged macrophages’ ability to promote angiogenesis

**Fig. 2:**
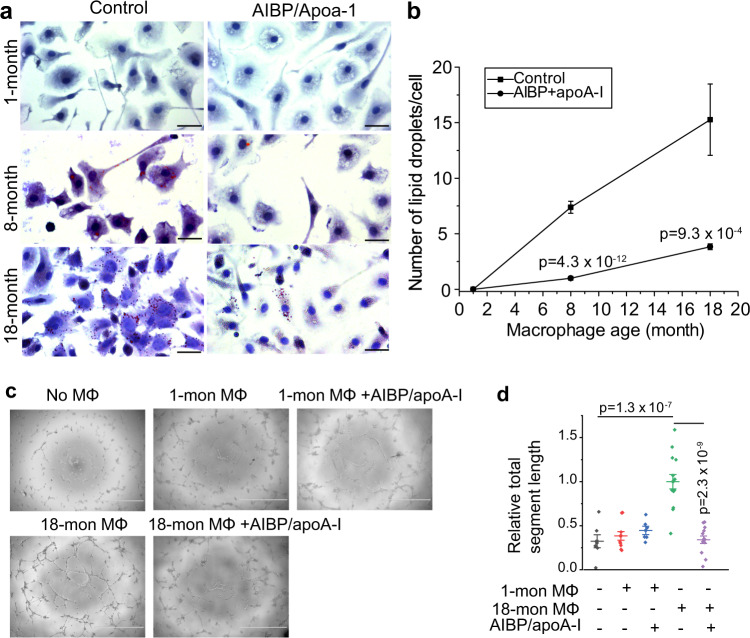
AIBP treatment reduces intracellular lipid accumulation in macrophages isolated from 8-month-old and 18-month-old mice and suppresses old macrophages’ ability to promote HRMEC angiogenesis. **a** Peritoneal macrophage isolated from 1-month, 8-month, and 18-month 57Bl6/J mice were treated with AIBP and apoA-I, and stained with oil red O. **b** Quantification on the number of lipid droplets per cell in macrophages. *N* = 25 (control) and 28 (AIBP/apoA-I) for 8-month mice, 43 (control) and 39 (AIBP/apoA-I) for 18-month mice. **c** Effect of AIBP on old macrophages’ ability to promote angiogenesis of HRMECs. Macrophages (MΦ) isolated from 1-month and 18-month mice were pretreated with different combination of AIBP and apoA-I and co-cultured with HRMECs. **d** Quantitative analysis of total segment length of tubes of HRMECs in **c**. *N* = 7 (without co-culture), 10 (1-month MΦ + control), 6 (1-month MΦ + AIBP/apoA-I), 14 (18-month MΦ + control), and 13 (18-month MΦ + AIBP/apoA-I) fields. Data represent mean ± SEM in **b**, **d**. Statistical analysis was performed by two-tailed Student’s *t*-test (**b**) or one-way ANOVA with Tukey post hoc analysis (**d**). Scale bar, 25 µm in **a**, 1000 µm in **c**.

Previous studies have shown that aged macrophages exhibit impaired cholesterol efflux, leading to intracellular lipid accumulation and pathologic vascular proliferation^8^. Indeed, we found that peritoneal macrophages isolated from 1-month, 8-month, and 18-month-old mice show an age-dependent increase of intracellular lipids as detected by oil red O staining (Fig. 2a, b). Consistent with recent data that AIBP effectively enhances cholesterol efflux from macrophages^14–16^, AIBP and apoA-I co-treatment markedly reduced lipid accumulation from macrophages that were isolated from 8-month and 18-month mice by 86.6% and 74.9%, respectively (Fig. 2b). Furthermore, AIBP and apoA-I co-treatment robustly inhibited old macrophages’ ability to promote angiogenesis of HRMECs co-cultured with peritoneal macrophages from 8-month (Supplementary Fig. 1) and 18-month (Fig. 2c, d) old mice. In contrast, AIBP and apoA-I co-treatment of young macrophages isolated from 1-month old mice had no effect on HRMEC angiogenesis. Our data suggest that AIBP/apoA-I treatment suppresses aged macrophages’ ability to promote angiogenesis.

### AIBP deficiency accelerates retinal and choroidal angiogenesis

**Fig. 3:**
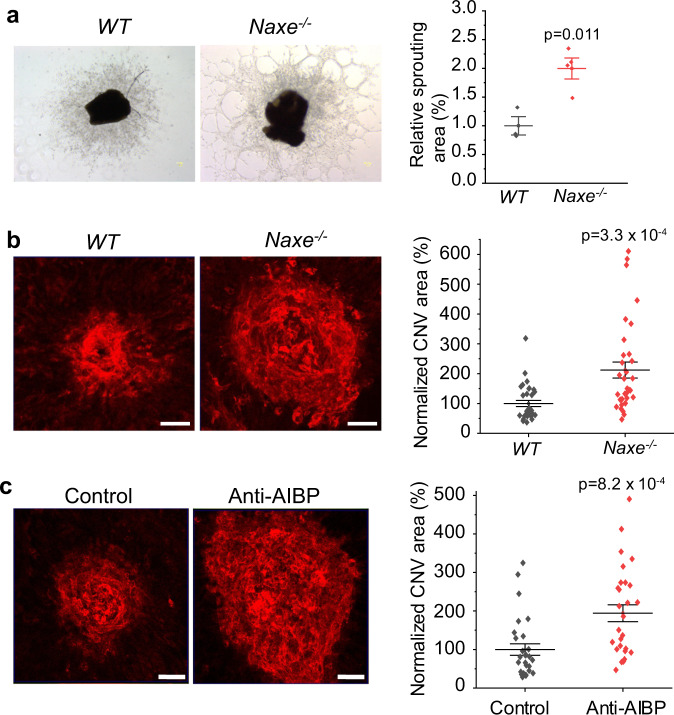
AIBP deficiency profoundly increases choroid sprouting and laser-induced CNV. **a** Representative images and quantification of microvascular sprouting area from *Naxe*^*−/−*^ and *WT* adult mouse choroid explants. *N* = 3 choroid explants for *WT*, 4 for *Naxe*^*−/−*^ mice. **b** Representative images of CNV lesions labeled by Alexa 568-isolectin on RPE-choroid flatmounts and quantification of CNV areas from *Naxe*^*−/−*^ and *WT* adult mice. *N* = 33 laser spots per group. **c** Representative images of CNV lesions and quantification of CNV areas after intravitreal injection of a rabbit anti-AIBP antibody or a control IgG. *N* = 28 laser spots per group. Scale bar is 20 µm in **b** and **c**. Data represent mean ± SEM in **a**–**c**. Statistical analysis was performed by two-tailed Student’s *t*-test.

AIBP deficiency was shown to markedly accelerate developmental retinal angiogenesis from P0 to P5^23^. To investigate the role of AIBP on choroid angiogenesis, we compared choroidal microvascular angiogenesis in ex vivo choroid explants isolated from *Naxe*^*−/−*^ (*Naxe* encoding AIBP) and WT mice. *Naxe*^*−/−*^ choroid explants exhibited a two-fold increase in sprouting area compared with WT controls (Fig. 3a). To examine the role of AIBP in CNV, we induced CNV by laser photocoagulation on WT and *Naxe*^*−/−*^ mice (2–3 months). One week after CNV induction, the CNV area was analyzed by Alexa 568-isolectin labeling on choroidal flatmounts. Loss of AIBP markedly increased the CNV area (2.1-fold, Fig. 3b). To test our hypothesis that the extracellular AIBP inhibits CNV, we used a rabbit polyclonal antibody (pAb) against AIBP^23^ to neutralize extracellular AIBP function. Preincubation of human AIBP protein with pAb abolished the inhibitory effect of AIBP on HRMEC angiogenesis, suggesting that the pAb antibody effectively neutralized extracellular AIBP function (Supplementary Fig. 2). We delivered 1.3 µg affinity purified pAb by intravitreal injection immediately after laser photocoagulation on WT mice. Consistent with the *Naxe*^*−/−*^ data ex vivo (Fig. 3a) and in vivo (Fig. 3b), AIBP neutralization caused a 1.9-fold increase of the CNV area (Fig. 3c), suggesting that extracellular AIBP inhibits pathogenic angiogenesis.

### AIBP expression is reduced in laser-induced murine CNV and human CNV

**Fig. 4:**
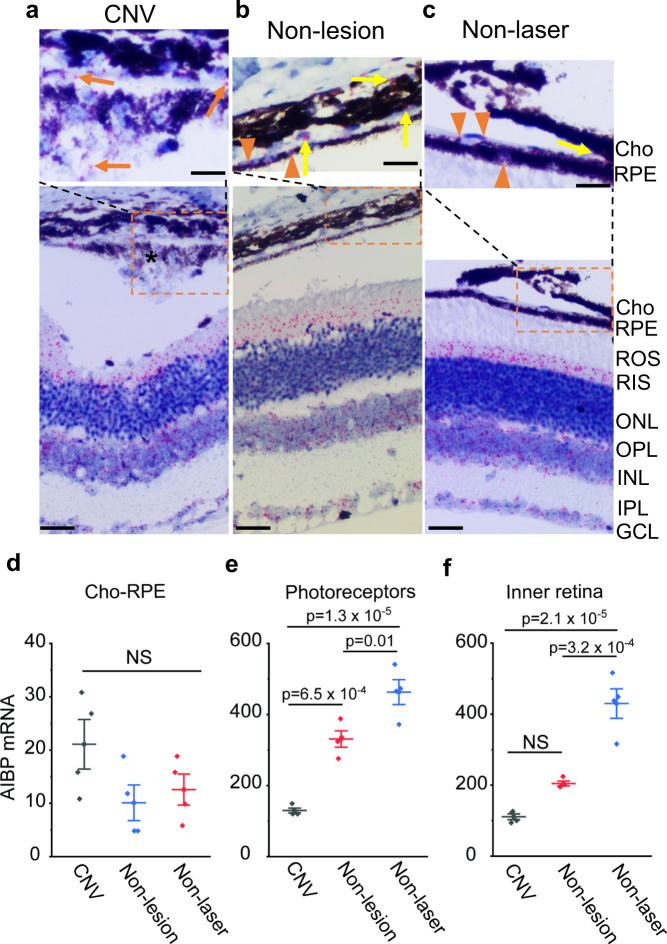
AIBP expression in mouse CNV, non-lesion, and control (non-laser) retinal areas. **a, b, c** AIBP mRNA (in red) detected by RNAscope counter stained by hematoxylin II in CNV, non-lesion, and non-laser retinal areas, repectively. The magnified images on the top show AIBP in RPE (orange arrowheads) and CNV membranes (orange arrows). Asterisk (*) indicates CNV membranes. Scale, 20 µm in magnified images and 40 µm in others. **d**, **e**, **f** Quantification of AIBP mRNA in the Cho-RPE, photoreceptors, and inner retina, respectively. *N* = 4 retinas for CNV (including CNV and non-lesion areas) and non-laser control. Data represent mean ± SEM. NS, *p* > 0.05. Statistical analysis was performed by one-way ANOVA with Tukey post hoc analysis. Cho choroid, ROS rod outer segment, RIS rod inner segment, ONL outer nuclear layer, OPL outer plexiform layer, INL inner nuclear layer, IPL inner plexiform layer, GCL ganglion cell layer.

To determine the AIBP expression in laser-induced CNV and human CNV specimens, we used RNAscope, which allows the detection of single mRNA transcripts in intact cells with high specificity^24^, to compare AIBP expression in CNV and non-lesion areas adjacent to CNV 7 days after laser injury, and in control retinas not subjected to laser injury (non-laser) in mice. In non-lesion and non-laser controls, AIBP was weakly expressed in the retinal pigment epithelium (RPE) (Fig. 4b, c, orange arrowheads) and choroid (Fig. 4b, c, yellow arrows). AIBP mRNA was mainly expressed in photoreceptors (inner segment, outer nuclear layer, and outer plexiform layer) and inner retina (inner nuclear layer, inner plexiform layer, and ganglion cell layer). AIBP was also weakly expressed in CNV membranes (Fig. 4a, orange arrows). AIBP expression is not significantly different between CNV, non-lesion and non-laser groups in the choroid-RPE (Fig. 4d). However, AIBP expression in photoreceptors was markedly reduced in CNV compared with both non-lesion and non-laser controls (Fig. 4e). AIBP expression in the inner retina in CNV was not significantly different from that in non-lesion areas (Fig. 4f), but was significantly reduced in CNV compared with the non-laser control (Fig. 4f, *p* = 2.1 × 10^–5^). In non-lesion areas adjacent to CNV, AIBP in both photoreceptors (*p* = 0.01) and inner retina (*p* = 3.2 × 10^–4^) was significantly reduced compared with the non-laser control. The likely reason for AIBP reduction in the non-lesion area is that laser induced CNV causes inflammation and other responses in the adjacent non-lesion areas. Photoreceptors produce the majority of AIBP in the outer retina (more than 20 times than that in RPE/choroid in normal, Fig. 4d, e), suggesting that the major source of AIBP in the outer retina that suppresses laser-induced CNV is produced by photoreceptors and secreted into the extracellular space. The drastic reduction of AIBP in photoreceptors in CNV contributes to CNV pathogenesis. In the negative control, no specific signal was detected in the *Naxe*^*−/−*^ retina, confirming the specificity of the *Naxe* probe (Supplementary Fig. 3).

**Fig. 5:**
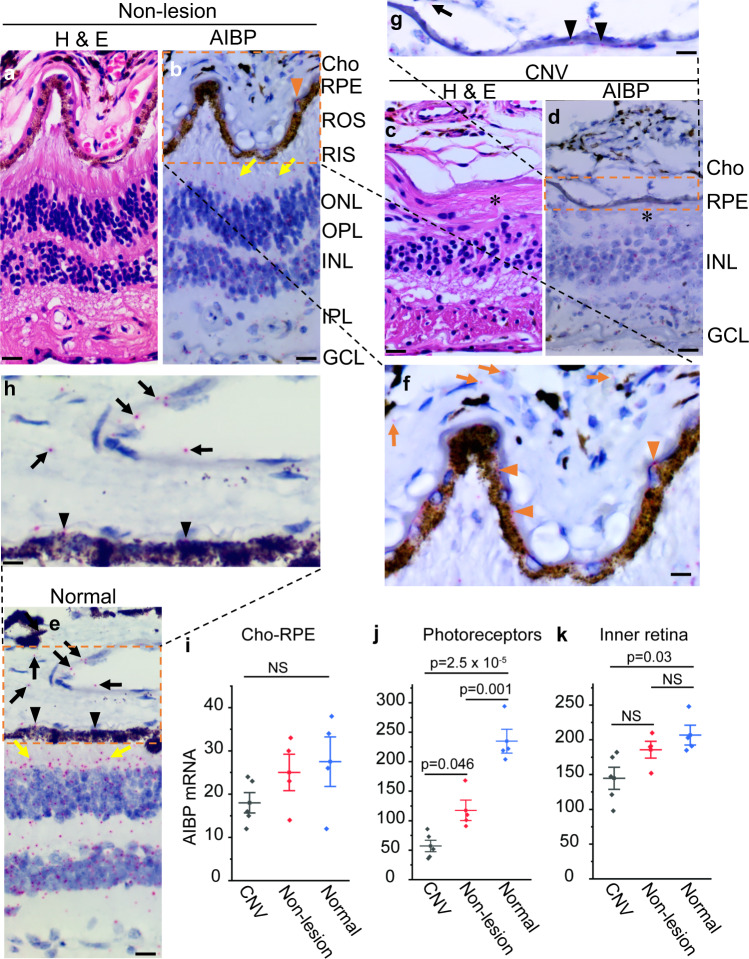
AIBP expression in human CNV, non-lesion, and normal control retinas detected by RNAscope. **a**, **c** H&E staining of non-lesion and CNV areas, respectively. **b**, **d**, **e** Representative RNAscope images of non-lesion, CNV, and normal retinas, respectively, show AIBP expression and localization. **a** and **c** are adjacent sections for **b** and **d**, respectively. **f, g, h** Magnified image of dashed orange box in **b**, **d**, and **e**, respectively. **i**, **j**, **k** Quantification of AIBP mRNA in Cho-RPE, photoreceptors, and inner retina, respectively. *N* = 5, 4, and 4 retinas for CNV, non-lesion areas, and normal, respectively. Data represent mean ± SEM. NS, *p* > 0.05. Statistical analysis was performed by one-way ANOVA with Tukey post hoc analysis. Yellow arrows, orange arrowheads, and orange arrows indicate AIBP expression in the inner segment, RPE, and choroid, respectively, in the normal and non-lesion areas. Black arrowheads and the black arrow indicate AIBP expression in the RPE and choroid, respectively, in normal and CNV areas. Asterisk (*) indicates subretinal disciform scar in CNV. Cho choroid, ROS rod outer segment, RIS rod inner segment, ONL outer nuclear layer, OPL outer plexiform layer, INL inner nuclear layer, IPL inner plexiform layer, GCL ganglion cell layer. Scale bar, 20 µm in **a**–**e**, 10 µm in **f**–**h**.

In a parallel study, we examined AIBP expression in human CNV. In normal and non-lesion areas adjacent to human CNV, AIBP was mainly expressed in photoreceptors (inner segment and outer nuclear layer) and inner neurons (inner nuclear layer, inner plexiform layer, and ganglion cell layer) (Fig. 5b, e, yellow arrows indicating AIBP expression in the inner segment). AIBP was weakly expressed in RPE (Fig. 5b, f, orange arrowheads; Fig. 5e, h, black arrowheads) and choroid (Fig. 5f, orange arrows; Fig. 5h, black arrows). This expression pattern is similar to that in the mouse retina (Fig. 4a). In CNV lesion areas, H & E staining revealed subretinal disciform scar and loss of photoreceptors and RPE over the scar (Fig. 5c, d, asterisk). AIBP was weakly expressed in choroid (Fig. 5g, black arrow) and degenerating RPE (Fig. 5g, black arrowheads). AIBP expression in choroid-RPE is not significantly different between CNV, non-lesion and normal groups (Fig, 5i). AIBP expression in photoreceptors was markedly reduced in CNV area (~76% reduction, *p* = 2.5 × 10^–5^) compared with normal (Fig. 5j). Since the AIBP reduction could be due to photoreceptor loss, we compared AIBP expression in non-lesion areas adjacent to CNV but with relatively intact photoreceptors with that in normal areas. AIBP expression was reduced by 51% (*p* = 0.046) even in non-lesion photoreceptors. Thus, AIBP reduction in photoreceptors of CNV lesion is the net result of photoreceptor loss and reduced expression, which is similar to that in laser-induced CNV (Fig. 4). AIBP expression in the inner retina in CNV was not significantly different from that in non-lesion areas, but was significantly reduced in CNV compared with the normal (Fig. 5k, *p* = 0.03). A negative control using a bacterial probe shows no signal (Supplementary Fig. 4). Since AIBP deficiency dramatically increases laser-induced CNV (Fig. 3b, c), marked AIBP reduction in the outer retina overlying CNV lesions is expected to exacerbate CNV.

### AIBP is superior to anti-VEGF agents in inhibiting CNV in 8-month mice

**Fig. 6:**
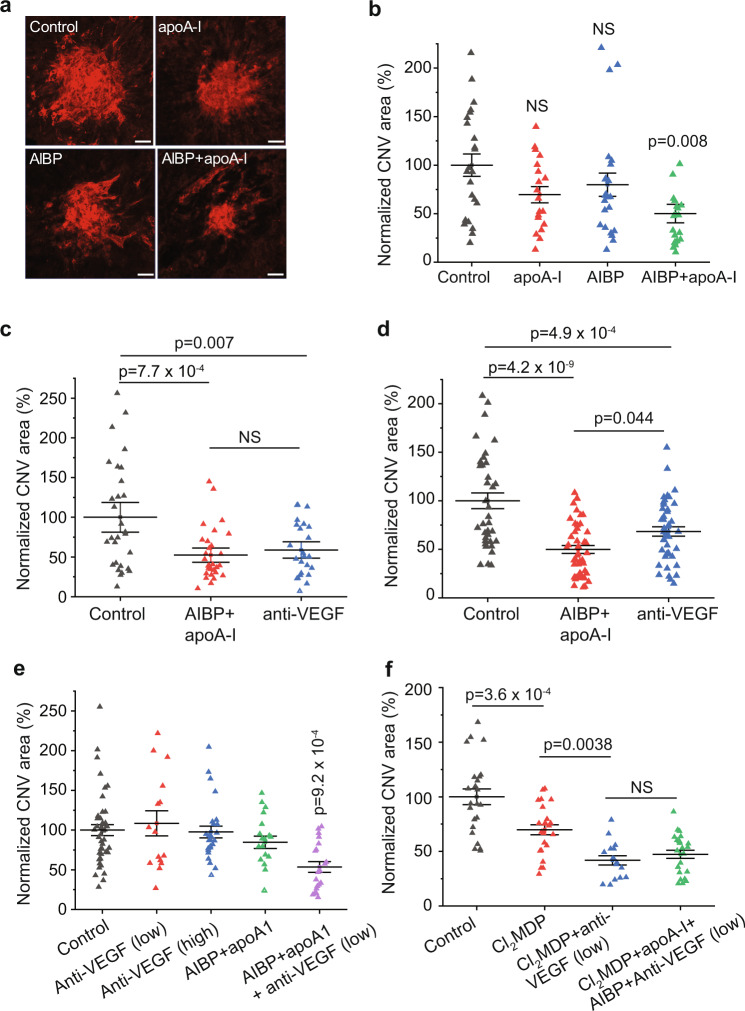
Comparison between AIBP/apoA-I, anti-VEGF agent, and combination treatment in suppressing laser-induced CNV in mice. **a** Representative images of CNV lesions after different combinations of AIBP and apoA-I treatment. **b**, Quantification of CNV areas in **a**. *N* = 23 (BSA control), 19 (apoA-I), 23 (AIBP), and 20 (AIBP + apoA-I) laser spots. **c**, **d** Comparison of AIBP/apoA-I and an anti-VEGF antibody in inhibiting laser-induced CNV in 6–8 weeks (**c**) and 8-month (**d**) mice. *N* = 29 (control), 30 (AIBP + apoA-I), and 24 (anti-VEGF) laser spots in **c**, and 37 (control), 47 (AIBP + apoA-I), and 43 (anti-VEGF) laser spots in **d**. **e** Quantification on the effect of anti-VEGF (low, 5 ng), anti-VEGF (high, 25 ng), AIBP + apoA-I, and AIBP + apoA-I + anti-VEGF (low) in suppressing laser-induced CNV in 18-month male mice. *N* = 43 (control), 15 (anti-VEGF low), 26 (anti-VEGF high), 19 (AIBP + apoA-I), and 20 (AIBP + apoA-I + anti-VEGF low) laser spots. **f** Macrophage depletion by Cl_2_MDP in old mice restored CNV sensitivity to anti-VEGF treatment and blunted the synergistic effect of combination therapy. *N* = 23 (control), 24 (Cl_2_MDP), 16 (Cl_2_MDP+anti-VEGF low), 24 (Cl_2_MDP+AIBP + apoA-I + anti-VEGF low) laser spots. Data represent mean ± SEM. NS, *p* > 0.05. Statistical analysis was performed by one-way ANOVA with Tukey post hoc analysis.

To test the efficacy of AIBP in treating CNV in vivo, we induced CNV in both eyes of WT mice (8–10 weeks) by laser photocoagulation. Immediately after laser delivery, mice received an intravitreal injection of AIBP alone, apoA-I alone, AIBP plus apoA-I, or BSA protein control. Seven days later, the eyes were collected and the CNV area was quantified in choroidal flatmounts. AIBP/apoA-I treatment reduced CNV area by 50.2% (*p* = 0.008) while AIBP or apoA-I alone non-significantly reduced CNV area by 21% and 31%, respectively (Fig. 6a, b). These results are consistent with our in vitro data showing that both AIBP and ApoA-I are necessary to inhibit angiogenesis (Fig. 1c).

To determine the optimal inhibiting dose of AIBP, we delivered different intravitreal injection doses of AIBP in combination with apoA-I (AIBP to apoA-I ratio was kept at 1 µg:4.2 µg) into the eyes of WT mice following the induction of CNV by laser photocoagulation. AIBP and apoA-I co-treatment inhibited laser-induced CNV in a dose-dependent manner (Supplementary Fig. 5a). We found that the 2.4 µg AIBP and 10 µg apoA-I combination was sufficient to produce maximal inhibition. To compare the efficacy of AIBP with anti-VEGF treatment, we first determined the dose-response curve of an anti-VEGF antibody (AF-493-NA, R&D Systems) given by intravitreal delivery at inhibiting laser-induced CNV. This anti-VEGF antibody dose-dependently inhibited CNV, and that 5 ng anti-VEGF antibody achieved maximal inhibition (Supplementary Fig. 5b). This amount was used to compare with that of the AIBP treatment (2.4 µg AIBP and 10 µg apoA-I combination). AIBP/apoA-I was equally effective as the anti-VEGF antibody at inhibiting laser-induced CNV in young mice (6–8 weeks) (Fig. 6c). Since AIBP targets both hyperactive VEGFR2 signaling in choroidal endothelial cells and intracellular lipid accumulation in old macrophages, two processes implicated in CNV pathogenesis, we tested the hypothesis that AIBP is more effective than anti-VEGF treatment in inhibiting CNV in older mice. Indeed, AIBP/apoA-I treatment reduced CNV area more than the anti-VEGF antibody in 8-month mice (49.9% vs. 68.4% CNV area, *p* = 0.044) (Fig. 6d). This is primarily due to the decreased efficacy of anti-VEGF antibody compared with that in young mice (68.4% vs. 51.8% CNV area, Fig. 6c, d).

### AIBP/apoA-I and anti-VEGF combination overcomes anti-VEGF resistance

We have shown age-dependent increase of intracellular lipids in macrophages (Fig. 2a, b). We thus expected that AIBP/apoA-I would be more effective than the anti-VEGF antibody in suppressing laser-induced CNV in 18-month than in 8-month old mice. However, neither AIBP nor anti-VEGF treatment was effective in inhibiting CNV in 18-month mice (Fig. 6e). No inhibition was observed even after we increased the amount of anti-VEGF antibody by five times (high, 25 ng) (Fig. 6e), which suggests that old mice are resistant to anti-VEGF treatment. Remarkably, the combination of AIBP/apoA-I and anti-VEGF antibody (low, 5 ng) overcame the anti-VEGF resistance and robustly suppressed laser induced CNV (46.5% reduction, *p* = 9.2 × 10^–4^).

We observed a similar effect by AIBP/apoA-I and anti-VEGF antibody in old female mice (14–15 months) (Supplementary Fig. 6). The synergistic effect is likely due to AIBP’s ability to disrupt VEGF-independent angiogenic pathways by regulating lipid rafts in endothelial cells and macrophages (see more below).

### Macrophage depletion restores CNV sensitivity to anti-VEGF treatment

The anti-VEGF resistance is likely caused by cholesterol-laden macrophages in old mice. To test this hypothesis, we used clodronate liposomes (Cl_2_MDP) to deplete macrophages in 18-month mice and performed laser-induced CNV. Macrophage depletion led to a 31% reduction in CNV lesion size, consistent with previous studies (Fig. 6f)^9,10^. Notably, the anti-VEGF antibody became effective at inhibiting CNV after macrophage depletion (40% reduction, *p* = 0.0038), suggesting that cholesterol-laden old macrophages play a key role in conferring anti-VEGF resistance in old mice. Furthermore, combination therapy of AIBP/apoA-I with the anti-VEGF antibody did not exhibit any synergistic effect after macrophage depletion (Fig. 6f). This experiment suggests that AIBP/apoA-I treatment augments the effectiveness of anti-VEGF therapy by targeting old macrophages. The most parsimonious explanation is that AIBP enhances cholesterol removal from old macrophages, which inhibits their ability to promote pathogenic angiogenesis (see Fig. 2c, d).

## Discussion

Anti-VEGF resistance remains a major challenge to current anti-VEGF therapy for CNV. Various strategies have been tested to overcome this issue, including increasing the frequency of anti-VEGF therapy, switching to different anti-VEGF agents, and combining anti-VEGF therapy with another treatment modality, e.g., photodynamic therapy. Various combination therapies are currently explored in clinical trials, e.g., targeting PDGF (Fovista) or the angiopoietin pathway. However, no major breakthrough has been reported. In fact, a phase III trial combining anti-VEGF and PDGF failed to demonstrate improved efficacy.

The mechanisms for anti-VEGF resistance remains elusive and the effort to develop new treatment is hampered in part, due to the lack of suitable AMD animal models that exhibit anti-VEGF resistance. By using mice from different ages, we demonstrated that laser-induced CNV in mice with increased age showed increased resistance to anti-VEGF treatment. In young mice (6–10 weeks), the most widely used age group due to their consistency and lack of variability in laser-induced CNV^25^, AIBP was equally effective as an anti-VEGF neutralizing antibody in suppressing laser-induced CNV. In the middle-aged group (8–10 months), the anti-VEGF agent was less effective whereas AIBP maintained the same efficacy as in treating the young mice. In old mice (>18 months), neither AIBP nor the anti-VEGF agent was effective in suppressing CNV. Remarkably, the combination of AIBP and anti-VEGF treatment overcomes the anti-VEGF resistance and effectively suppresses CNV. Several lines of evidence suggest that the accumulation of intracellular lipids in old macrophages plays a critical role in anti-VEGF resistance in this model. First, the decrease in efficacy of anti-VEGF therapy with age is inversely correlated with the age-dependent increase of intracellular lipids in macrophages. Second, macrophage depletion in old mice restores CNV sensitivity to anti-VEGF treatment. Third, the beneficial effect of AIBP is likely due to both its ability to enhance cholesterol efflux from macrophages and its anti-inflammatory function^14–16^. Further studies are necessary to determine the molecular mechanism(s) connecting aged macrophages and anti-VEGF resistance.

CNV is a process that involves both angiogenesis and inflammation^26^. Our data suggest that the VEGF-dependent angiogenic pathway plays a dominant role in CNV pathogenesis in young mice (6–10 weeks). However, with aging, alternative angiogenic pathways involving cholesterol-laden macrophages and endothelial cells exert increasingly larger roles in CNV, leading to resistance to anti-VEGF monotherapy. Previous studies have shown that VEGF165 acts as a proinflammatory cytokine targeting monocytes, macrophages, and leukocytes in a positive feedback loop involving primarily endothelial cells to sustain pathological neovascularization^11,12^. This may explain why both the neutralization of extracellular VEGF by anti-VEGF agents and the enhanced removal of cholesterol from old macrophages by AIBP/apoA-I are required to cut off the vicious cycle between endothelial cells and macrophages to combat anti-VEGF resistance. Furthermore, both *Naxe*^*−/−*^ and AIBP neutralization data showed that AIBP plays a critical role in regulating pathogenic angiogenesis (i.e., CNV) likely by enhancing cholesterol efflux from both endothelial cells and macrophages (Fig. 3b, c). Marked AIBP reduction in the outer retina in human CNV lesions (Fig. 5) is expected to exacerbate CNV in human neovascular AMD, and contribute to CNV pathogenesis. We speculate that AIBP in the outer retina, which is mainly produced by photoreceptors, plays an important role in inhibiting the progression of choroidal NV in subretinal space while AIBP in both inner and outer retina may play a role in inhibiting type 3 NV. Thus, delivery of exogenous AIBP/apoA-I is a promising treatment that could reduce CNV or overcome anti-VEGF resistance for patients with neovascular AMD.

Substantial evidence indicate that macrophages have an important role in the pathogenesis of wet AMD in both animal models and human patients^8–10,27–33^. Oxidized low-density lipoprotein and macrophages have been detected in CNV membranes from eyes with AMD^34^. In particular, macrophage density and the proliferation of infiltrated inflammatory cells are increased in CNV membranes from patients previously treated with Avastin^7^, which implies a mechanism for tachyphylaxis to anti-VEGF treatment. Our study provides strong evidence that cholesterol-laden macrophages confer anti-VEGF resistance in wet AMD and that combination of anti-VEGF agents and AIBP/apoA-I can be a potential therapeutic solution. Since anti-VEGF therapy has become the mainstay of treatment for a plethora of ocular pathologies including CNV, diabetic retinopathy, and retinal vein occlusion, etc., this work will have broad implications in the clinic in treating patients that are not benefiting from the current therapy. Although the laser-induced model of CNV does not have the age-related progressive pathology in AMD, it captures many of the important features of the human condition (e.g., newly formed vessels arise from the choroid and invade into the subretinal space, accumulation of macrophages near arborizing neovascular membranes^35–38^, etc.). This model has been successful in predicting the clinical efficacy of anti-VEGF therapy for neovascular AMD^39^. It is also widely used for studying CNV and assessing anti-angiogenic drugs in vivo. The involvement of macrophages in anti-VEGF resistance in this model and in human AMD^7^ suggest the validity of this model to study the mechanism and treatment strategies of anti-VEGF resistance. Since there are no animal models recapitulating all features of neovascular AMD, the efficacy of AIBP/apoA-I/anti-VEGF combination therapy in overcoming anti-VEGF resistance in human AMD ultimately needs to be evaluated in human clinical trials.

## Methods

### Mice

WT (*C57BL/6J*) mice were purchased from Jackson Laboratory. Old male C57BL/6 mice (18 months) were ordered from Jackson Laboratory or National Institute of Aging. Old female C57BL/6 mice (14–15 months) were ordered from the Comparative Medicine of Baylor College of Medicine or bred from Jackson mice. *Naxe*^*−/−*^ mice were generated previously^23^. All animal experiments were approved by the Institutional Animal Care and Use Committees (IACUC) at Baylor College of Medicine, Houston, and Houston Methodist Research Institute, Houston.

### Cells

Primary human retinal microvascular endothelial cells (HRMECs) were purchased from Cell Systems Corporation (Kirkland, WA, USA). More than 95% of the cells are CD31 and VWF positive, and take up Dil-LDL, but are negative for NG2 and PDGFRb. HRMECs were cultured at 37 °C with 5% CO2 in a humid atmosphere in Endothelial Basal Medium (EBM-2) with 2% fetal bovine serum (FBS). Peritoneal macrophages recruitment was elicited by intraperitoneal injection of 4% thioglycollate. Five days after injection, macrophages were harvested and cultured in DMEM/F12 with 10% FBS, 100 U/mL penicillin, and 100 μg/mL streptomycin at 37 °C overnight^40^. Macrophages were then washed with PBS and non-adherent cells were removed.

### D4-GFP staining to assess cell membrane cholesterol content

1.5 × 10^4^ HRMECs were placed on 0.2% gelatin-coated coverglasses in a 24-well plate. On the following day, HRMECs were treated with control base medium, recombinant AIBP (100 ng/mL), HDL_3_ (50 µg/mL), or combination (100 ng/mL AIBP and 50 µg/mL HDL_3_) conditions in EBM-2 supplemented with 0.1% BSA for 4 h in a 37 °C incubator. After washing with PBS for 3 times, cells were incubated with recombinant D4-GFP (50 μg/mL) in EBM-2 supplemented with 0.1% BSA for 1.5 h on ice. After washing with cold PBS 3 times, cells were fixed with 4% paraformaldehyde 10 min at room temperature and mounted for imaging with a Leica epifluorescence microscope (Leica DM4000). GFP positive areas per cell were quantified using ImageJ.

### Tube formation assay

HRMECs were seeded in each well of a 96-well plate, which were coated with 1:1 mix of Matrigel (Corning, USA) and EBM-2, at a density of 1 × 10^4^ cells/well in 200 μL EBM-2. For HRMEC-macrophage co-culture, peritoneal macrophages were preincubated with 0.2 µg/mL AIBP and 25 µg/mL apoA-I (individually or in combination) for 4 h^23^. HRMECs and peritoneal macrophage were then mixed (10:1 ratio) and seeded on growth factor-reduced Matrigel mixed 1:1 with EBM-2 in 96-well culture plate. Cells were incubated at 37 °C for 4–6 h before being imaged by a light microscope. The total segment length or tube length was quantified using ImageJ.

### Choroidal sprouting assay

Choroidal sprouting experiments were performed as previously described^21^. The RPE/choroid from 4-week old C57BL/6 J or *Naxe*^*−/−*^ mice were cut into approximately 1 × 1 mm^2^ pieces, and placed in growth factor-reduced Matrigel in 24-well plates with endothelial cell ECM complete medium at 37 °C. For explants treated with AIBP/apoA-I, 0.2 µg/mL AIBP and 25 µg/mL apoA-I (alone or in combination) were added to the medium and incubated for 4 h every 2 days. Images of individual explants were taken 4–5 days after embedding and the area of microvascular sprouting was quantified using ImageJ.

### Oil red O staining

Peritoneal macrophages were washed twice with cold phosphate-buffered saline (PBS) and fixed with 4% paraformaldehyde (PFA) for 10 min at 37 °C. Cells were placed in 100% propylene glycol and incubated for 10 min at room temperature with occasional shaking. Cells were then incubated with pre-warmed 0.5% oil red O at 65 °C for 10 min with occasional stirring. After removing the oil red O solution, cells were incubated with 75% propylene glycol for 5 min at room temperature, washed with H_2_O, and counterstained with hematoxylin prior to examination by microscopy.

### Laser-induced choroidal neovascularization (CNV)

Laser photocoagulation was carried out as described previously using the Micron IV retinal imaging system (Phoenix Research Lab, Pleasanton, CA, USA) with the Meridian Merilas 532 green laser (240 mW, 200 ms, 50 μm spot)^25^. *C57BL/6J* mice (males and females: 6–8 weeks and 2–3 months; 8–10-month-old males, 18-month-old males, and 14–15-month-old females) and *Naxe*^*−/−*^ mice (2–3 months, males and females) were used. Slightly younger female mice were used in the old mouse group because old female mice showed more severe CNV than males^41,42^. At 1 week after laser treatment, CNV was analyzed in choroidal flat mounts by Alexa 568 isolectin B4 staining and lesion size was quantified by ImageJ.

### Intravitreal delivery

Intravitreal injection in mice was performed as previously described^43^ with an injection volume of 1.2 μL. For most experiments, 2.4 µg AIBP, 10 µg apoA-I, 5 ng anti-VEGF antibody (AF-493-NA, R&D Systems), 12.4 µg BSA, and 5 ng purified goat IgG (control for anti-VEGF antibody) were delivered individually or in combination unless otherwise indicated. For antibody neutralization of AIBP, 1.3 µg affinity purified rabbit anti-AIBP polyclonal antibody^23^ was delivered by intravitreal injection immediately after laser photocoagulation to WT mice. The anti-AIBP antibody was validated using *Naxe*^*−/−*^ and *WT* mice by western blot.

### Human specimens

Human CNV specimens were obtained from three patients: one 75-year-old man, one 80-year-old man, and one 90-year-old man. All patients were of Caucasian ethnicity with neovascular AMD. Three control eye specimens were obtained from 57–80-year-old Caucasian donors without AMD. The use of the postmortem human donor eyes was approved by the Institutional Review Board (IRB) at Baylor College of Medicine, Houston, and Johns Hopkins University School of Medicine, Baltimore.

### RNAscope

Retina sections of human or mouse eyes were used for RNAscope assay to detect AIBP mRNA expression and localization. Tissues were hybridized with target oligo probes (Advanced Cell Diagnostics, Newark, CA) for mouse or human AIBP, or a negative control probe targeting bacterial dihydrodipicolinate reductase. The AIBP was detected with the RNAscope Fluorescent Multiplex Kit or RNAscope 2.5 HD Chromogenic Detection Kit (Advanced Cell Diagnostics) according to the manufacturer’s protocol with the following modifications: (1) For the fluorescent RNAscope assay with mouse eyes, tissues were post-fixed in 4% PFA for 90 min at room temperature to preserve tissue integrity after baking slides for 30 min at 60 °C; (2) For the chromogenic RNAscope assay with human eyes, target retrieval duration was set at 10 min and the amplification step 5 was doubled to 60 min. Images were collected with an Olympus BX53 microscope (for Fast Red detection) or Zeiss LSM800 Confocal microscope (for fluorescent detection). RNA numbers were counted manually.

### Macrophage depletion

Mice were anesthetized by intraperitoneal injection of ketamine/xylazine (70–100/10–20/kg body weight). Splenic and systemic macrophage depletion was performed with 150 µL Clodrosome (18.4 mM CL_2_MDP, Encapsula NanoSciences LLC, Brentwood, TN) by intraperitoneal (IP) administration 3 days and 24 h before the laser procedure and 3 days after the laser procedure. Control group received IP administration of PBS.

### Statistics and reproducibility

No statistical methods were used to predetermine sample size. Sample size was based on experimental feasibility, sample availability, and N necessary to obtain definitive, significant results. For intravitreal injection and laser-induced CNV, some animals were excluded from the experiments due to severe hemorrhage and animal death. Unsuccessful laser burns without Bruch’s membrane rupture were excluded from laser-induced CNV. For in vivo studies in mice (young and old), both male and female mice were randomly distributed into different treatment groups. Researchers were not blinded to animal group allocation. All group results are expressed as mean ± SEM. Measurements were taken from distinct samples. Statistical comparisons were made using the two-tailed Student’s *t*-test for two groups or one-way analysis of variance (ANOVA) with Tukey’s post hoc for multiple groups. Values of ‘*N*’ were described in figure legends. Statistical significance (*p* value) was indicated in the figures and figure legends. Statistical analysis was performed with OriginPro or GraphPad Prism. All relevant data are included in the manuscript and/or in supplementary information files.

### Reporting summary

Further information on research design is available in the Nature Research Reporting Summary linked to this article.

## Supplementary information

Supplementary Data 1

Description of Additional Supplementary Files

Reporting Summary

Supplementary Information

## Data Availability

The source data for the graphs in the main figures are included in Supplementary Data 1. Other original data that support the findings of this study are available on reasonable request to the corresponding authors (Y.F. or L.F.). Unique materials will be made available to investigators at academic institutions for non-commercial research upon signing an MTA if applicable.
