# Supplementary material for: Combination of apolipoprotein-A-I/apolipoprotein-A-I binding protein and anti-VEGF treatment overcomes anti-VEGF resistance in choroidal neovascularization in mice
Source: Commun Biol. 2020 Jul 16;3:386. doi: 10.1038/s42003-020-1113-z (PMC7367303; doi:10.1038/s42003-020-1113-z)
Supplement: Supplementary file 2 — Description of Additional Supplementary Files [file 42003_2020_1113_MOESM2_ESM.pdf]

Description of Additional Supplementary Files

File Name: **Supplementary Data 1**
Description: The source data for the graphs in the main figures
