# Supplementary material for: Combination of apolipoprotein-A-I/apolipoprotein-A-I binding protein and anti-VEGF treatment overcomes anti-VEGF resistance in choroidal neovascularization in mice
Source: Commun Biol. 2020 Jul 16;3:386. doi: 10.1038/s42003-020-1113-z (PMC7367303; doi:10.1038/s42003-020-1113-z)
Supplement: Supplementary file 3 — Reporting Summary [file 42003_2020_1113_MOESM3_ESM.pdf]

natureresearch

Corresponding author(s): Yingbin Fu, Longhou Fang

Last updated by author(s): Jun 10, 2020

Reporting Summary

Nature Research wishes to improve the reproducibility of the work that we publish. This form provides structure for consistency and transparency in reporting. For further information on Nature Research policies, see Authors & Referees and the Editorial Policy Checklist.

Statistics

For all statistical analyses, confirm that the following items are present in the figure legend, table legend, main text, or Methods section.

n/a	Confirmed	
☐	☒	The exact sample size (*n*) for each experimental group/condition, given as a discrete number and unit of measurement
☐	☒	A statement on whether measurements were taken from distinct samples or whether the same sample was measured repeatedly
☐	☒	The statistical test(s) used AND whether they are one- or two-sided<br>*Only common tests should be described solely by name; describe more complex techniques in the Methods section.*
☒	☐	A description of all covariates tested
☒	☐	A description of any assumptions or corrections, such as tests of normality and adjustment for multiple comparisons
☐	☒	A full description of the statistical parameters including central tendency (e.g. means) or other basic estimates (e.g. regression coefficient) AND variation (e.g. standard deviation) or associated estimates of uncertainty (e.g. confidence intervals)
☐	☒	For null hypothesis testing, the test statistic (e.g. *F*, *t*, *r*) with confidence intervals, effect sizes, degrees of freedom and *P* value noted<br>*Give P values as exact values whenever suitable.*
☒	☐	For Bayesian analysis, information on the choice of priors and Markov chain Monte Carlo settings
☒	☐	For hierarchical and complex designs, identification of the appropriate level for tests and full reporting of outcomes
☒	☐	Estimates of effect sizes (e.g. Cohen's *d*, Pearson's *r*), indicating how they were calculated

Our web collection on statistics for biologists contains articles on many of the points above.

Software and code

Policy information about availability of computer code

Data collection	Brightfield images were collected with an Olympus BX53 microscope. Fluorescent images were collected with Zeiss LSM800 Confocal microscope or Leica DM4000 epifluorescence microscope
Data analysis	Quantification of images was performed using ImageJ. RNAs were counted manually for RNAscope assay.

For manuscripts utilizing custom algorithms or software that are central to the research but not yet described in published literature, software must be made available to editors/reviewers. We strongly encourage code deposition in a community repository (e.g. GitHub). See the Nature Research guidelines for submitting code & software for further information.

Data

Policy information about availability of data

All manuscripts must include a data availability statement. This statement should provide the following information, where applicable:

- Accession codes, unique identifiers, or web links for publicly available datasets
- A list of figures that have associated raw data
- A description of any restrictions on data availability

The source data for the graphs in the main figures are included in Supplementary Data 1. Other original data that support the findings of this study are available on reasonable request to the corresponding author (Y.F. or L.F.). Unique materials will be made available to investigators at academic institutions for non-commercial research upon signing an MTA if applicable.(Y.F. or L.F.).

Field-specific reporting

Please select the one below that is the best fit for your research. If you are not sure, read the appropriate sections before making your selection.

☒ Life sciences ☐ Behavioural & social sciences ☐ Ecological, evolutionary & environmental sciences

For a reference copy of the document with all sections, see nature.com/documents/nr-reporting-summary-flat.pdf

Life sciences study design

All studies must disclose on these points even when the disclosure is negative.

Sample size	No statistical methods were used to predetermine sample size. Sample size was based on experimental feasibility, sample availability, and N necessary to obtain definitive, significant results.
Data exclusions	For intravitreal injection and laser-induced CNV, some animals were excluded from the experiments due to severe hemorrhage and animal death. Unsuccessful laser burns without Bruch's membrane rupture were excluded from laser-induced CNV.
Replication	The experimental findings were reliably reproduced through repeated experiments.
Randomization	For in vivo studies in mice (young and old), both male and female mice were randomly distributed into different treatment groups.
Blinding	Researchers were not blinded to animal group allocation.

Reporting for specific materials, systems and methods

We require information from authors about some types of materials, experimental systems and methods used in many studies. Here, indicate whether each material, system or method listed is relevant to your study. If you are not sure if a list item applies to your research, read the appropriate section before selecting a response.

Materials & experimental systems

n/a	Involved in the study
☐	☒ Antibodies
☐	☒ Eukaryotic cell lines
☒	☐ Palaeontology
☐	☒ Animals and other organisms
☒	☐ Human research participants
☒	☐ Clinical data

Methods

n/a	Involved in the study
☒	☐ ChIP-seq
☒	☐ Flow cytometry
☒	☐ MRI-based neuroimaging

Antibodies

Antibodies used	custom-made rabbit anti-AIBP antibody
Validation	The anti-AIBP antibody was validated using Apoa1bp-/- and WT mice by western blot.

Eukaryotic cell lines

Policy information about cell lines

Cell line source(s)	Primary human retinal microvascular ECs (HRMECs) were purchased from Cell Systems, Kirkland, WA, USA (ACBRI 181)
Authentication	More than 95% of the cells are CD31 and VWF positive, and take up Dil-LDL, but are negative for NG2 and PDGFRb.
Mycoplasma contamination	All cell lines were routinely tested for mycoplasma. Cell lines that tested positive were discarded and the collected data (if any) was not used in the analyses.
Commonly misidentified lines (See ICLAC register)	None used.

Animals and other organisms

Policy information about studies involving animals; ARRIVE guidelines recommended for reporting animal research

Laboratory animals	WT (C57BL/6J) mice were purchased from Jackson Laboratory. Old C57BL/6 mice (18 months) were ordered from Jackson Laboratory or National Institute of Aging. Old female C57BL/6 mice (14-15 months) were ordered from the Comparative Medicine of Baylor College of Medicine or bred from Jackson mice. Naxe-/- mice were originally generated at Yury Miller's lab at UCSD. All mice were maintained in a specific pathogen-free facility. All animal experiments were approved by the Institutional Animal Care and Use Committees (IACUC) at Baylor College of Medicine, Houston, and Houston Methodist Research Institute, Houston.
Wild animals	Wild animals were not used in the study
Field-collected samples	This study did not involve samples collected from the field.
Ethics oversight	Institutional Animal Care and Use Committees (IACUC) at Baylor College of Medicine and Houston Methodist Research Institute.

Note that full information on the approval of the study protocol must also be provided in the manuscript.
