# Supplementary material for: Combination of apolipoprotein-A-I/apolipoprotein-A-I binding protein and anti-VEGF treatment overcomes anti-VEGF resistance in choroidal neovascularization in mice
Source: Commun Biol. 2020 Jul 16;3:386. doi: 10.1038/s42003-020-1113-z (PMC7367303; doi:10.1038/s42003-020-1113-z)
Supplement: Supplementary file 4 — Supplementary Information [file 42003_2020_1113_MOESM4_ESM.pdf]

Supplementary Figures

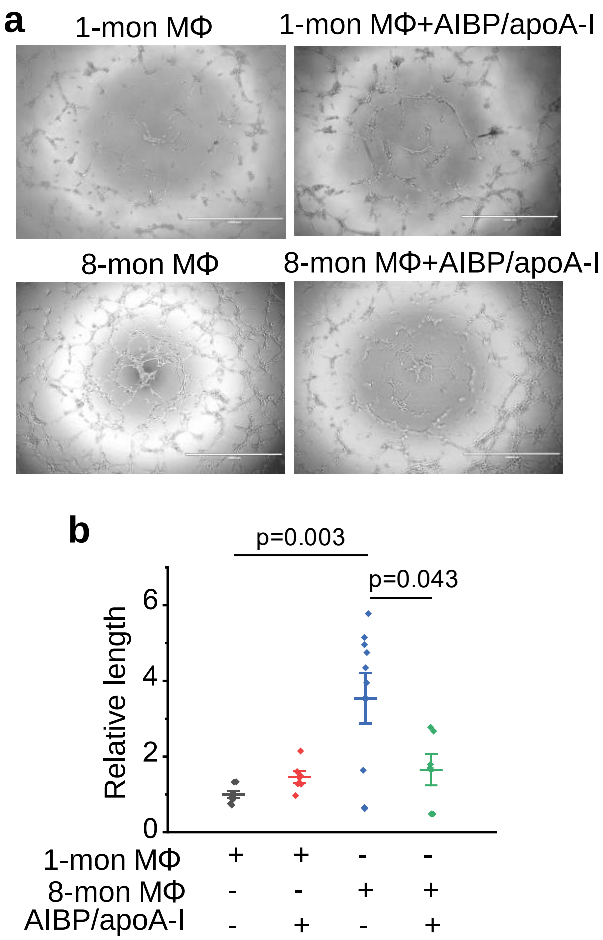

Supplementary Fig. 1. AIBP suppresses old macrophage's ability to promote angiogenesis of HRMECs. Representative images **(a)** and quantitative analysis **(b)** of total segment length of HRMECs co-cultured with peritoneal macrophages isolated from 1-month and 8-month mice. Macrophages were pretreated with different combinations of AIBP and apoA-I. N=7 (1-month MΦ + control), 6 (1-month MΦ + AIBP/apoA-I), 9 (8-month MΦ + control), and 6 (8-month MΦ +AIBP/apoA-I). Data represent mean ± SEM. Statistical analysis was performed by one-way ANOVA with Tukey *post hoc* analysis. Scale bar, 1000 µm in **a**.

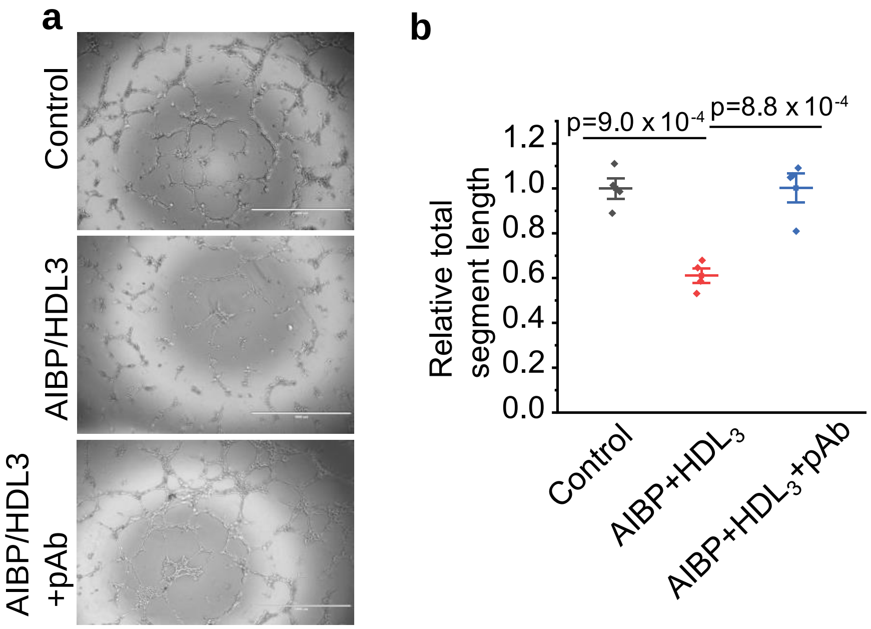

Supplementary Fig. 2. AIBP neutralization abolishes the inhibitory effect of AIBP on HRMEC angiogenesis. (a) Effect of AIBP pAb antibody neutralization on the inhibitory effect of AIBP on HRMEC tube formation. Scale bar, 1000 µm. **(b)** Quantification of the total tube length. N=4 per group. Data represent mean ± SEM. Statistical analysis was performed by one-way ANOVA with Tukey *post hoc* analysis. pAb, polyclonal antibody.

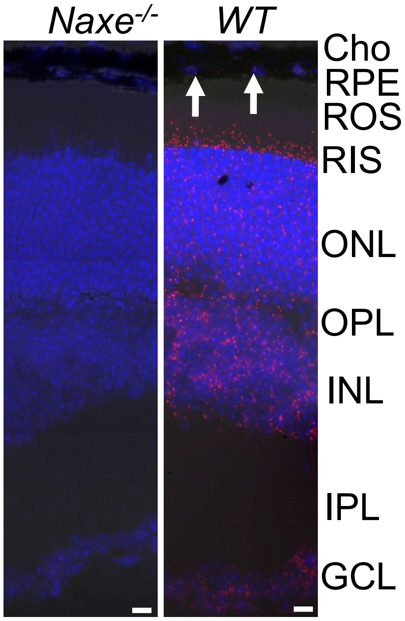

Supplementary Fig. 3. AIBP expression in the retina of WT and *Naxe*$^{-/-}$ mice. Robust AIBP mRNA can be detected in WT retina, but not in *Naxe*$^{-/-}$ retina. White arrows indicate weak AIBP expression in the RPE of *WT* mice. AIBP mRNA was detected by RNAscope (in red). Nuclei were labeled with DAPI (blue). Fluorescent images were overlaid with brightfield to show RPE and choroid. Scale bar, 10 μm.

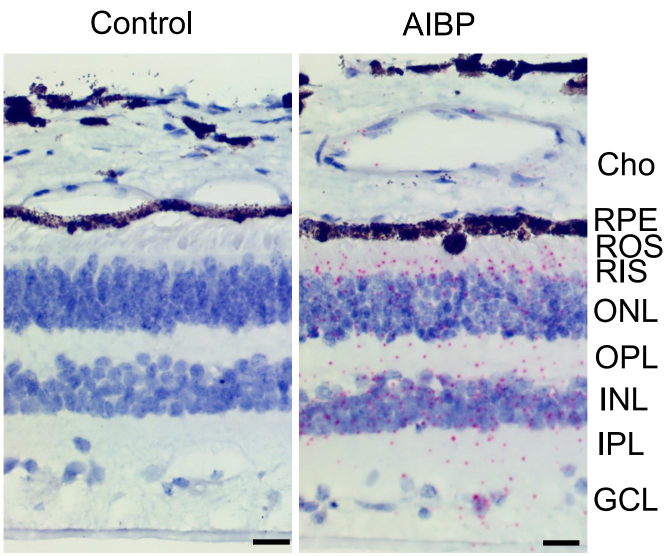

Supplementary Fig. 4. A negative control probe targeting bacterial dihydrodipicolinate reductase shows no signal on human retinal section. Human retinal paraffin sections were probed with either the negative control probe against bacterial dihydrodipicolinate reductase or the AIBP probe. Scale bar, 20 µm.

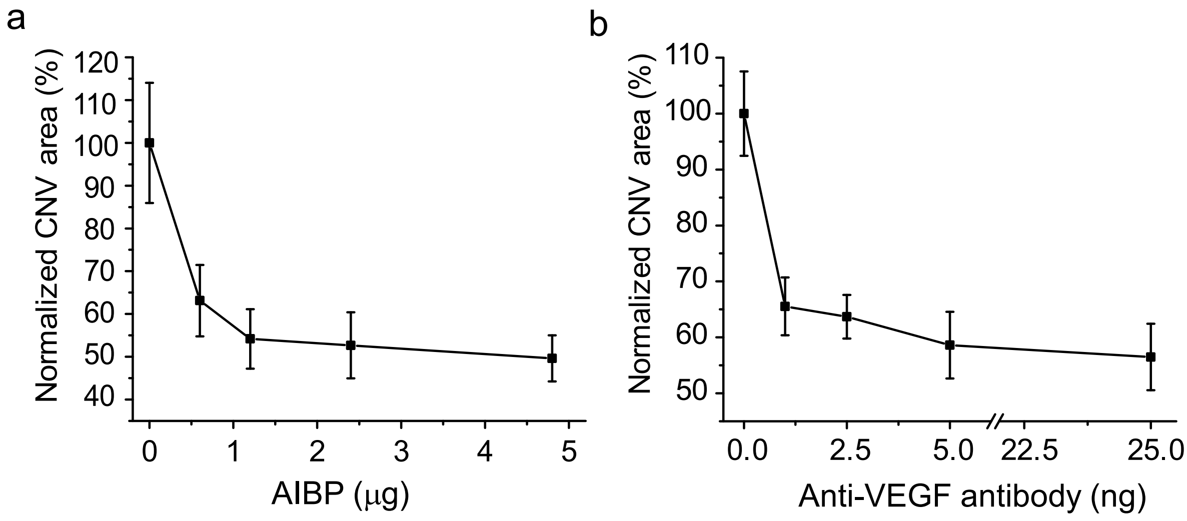

Supplementary Fig. 5. Dose-range study for AIBP and anti-VEGF in inhibiting laser-induced CNV. a, Dose-ranging study for AIBP and apoA-I. N=21-30 per dose. **b,** Dose-ranging study for an anti-VEGF neutralizing antibody. N=25-49 per dose. Data represent mean ± SEM.

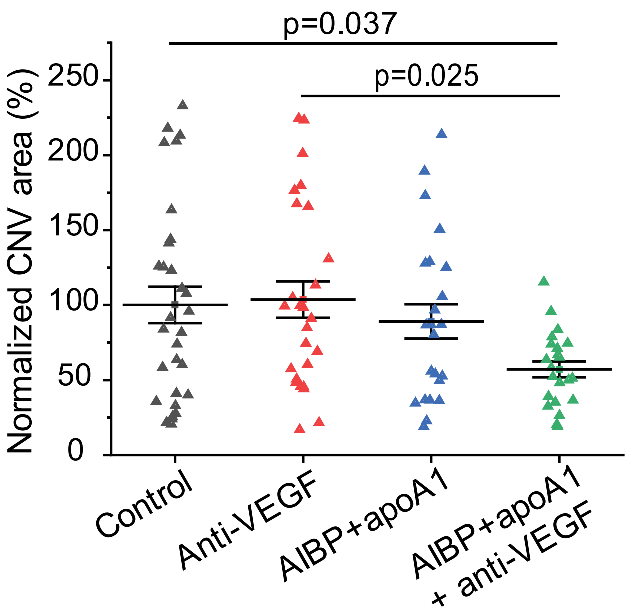

Supplementary Fig. 6. Comparison between AIBP/apoA-I, anti-VEGF agent, and combination treatment in suppressing laser-induced CNV in old female mice. Quantification on the effect of anti-VEGF (5 ng), AIBP+apoA-I, and AIBP+apoA-I+anti-VEGF in suppressing laser-induced CNV in 14-15 months female mice. N=30 (BSA control), 26 (anti-VEGF), 23 (AIBP+apoA-I), and 22 (AIBP+apoA-I+anti-VEGF) laser spots. Data represent mean ± SEM. Statistical analysis was performed by one-way ANOVA with Tukey *post hoc* analysis.
